# On the Iodine of the Atmosphere

**Published:** 1852-04

**Authors:** 


*On the Iodine of the Atmosphere.* By M. Chatin.—The constant dispersion of iodine, through the slow, spontaneous evaporation of the waters which contain it, and its more rapid volatilisation when heat is applied to these; its elimination from hard ward waters, which is so speedy that it can seldom be detected therein, even when they spring from highly iodined soils; and the results, though incomplete, which have been obtained by operating on rain water, are so many circumstances which have led M. Chatin to conclude that this substance must exist in the atmosphere. He estimates the 4000 *litres* of air, which traverse the lungs of a man in 12 hours, as containing 1–45 milligramme, *i. e.*, the same quantity that is found in a *litre* of potable water moderately iodined. This iodine becomes fixed dnring the act of respiration, the expired gases exhibiting about 1–5 of the iodine contained in the inspired air. The atmosphere of ill-ventilated and crowded places is in part deprived of its iodine. The proportion of iodine contained in the waters of a given locality indicates approximatively the quantity contained in its atmosphere. Rain is notably more iodined in the interior than in the vicinity of the coast, inasmuch as the iodine of fresh waters is much more completely dispersed than is that of sea-water. Great differences, due to causes not yet appreciated, exist in the amount of iodine contained in the rain of the same locality; the proportion, however, always diminishing when the rains are very prolonged. As rain always loses its iodine on falling, this might be fixed for useful purposes by placing in cisterns a millionth or a half-millionth part of carbonate of potash. Snow is iodined; but, *cateris paribus*, less so than rain. Dew contains iodine. Additional observations are required to decide whether iodine exists in the air in the free state, as hydriodic acid, as hydriodate of ammonia, or as forming a volatile combination with certain organic elements. *Gaz. Med.*, 1851, No. 19, p. 300.

